# Relatives’ attachment anxiety mediates the association between perceived loss and expressed emotion in early psychosis

**DOI:** 10.1371/journal.pone.0223425

**Published:** 2019-10-07

**Authors:** Lídia Hinojosa-Marqués, Tecelli Domínguez-Martínez, Tamara Sheinbaum, Paula Cristóbal-Narváez, Thomas R. Kwapil, Neus Barrantes-Vidal

**Affiliations:** 1 Departament de Psicologia Clínica i de la Salut, Universitat Autònoma de Barcelona, Barcelona, Spain; 2 Centro de Investigación en Salud Mental Global, Dirección de Investigaciones Epidemiológicas y Psicosociales, Instituto Nacional de Psiquiatría “Ramón de la Fuente Muñiz”, Mexico City, Mexico; 3 Department of Psychology, University of Southern California, Los Angeles, California, United States of America; 4 Unitat de Recerca, Docència i Innovació, Parc Sanitari Sant Joan de Déu, San Boi de Llobregat, Barcelona, Spain; 5 Department of Psychology, University of Illinois at Urbana-Champaign, Champaign, Illinois, United States of America; 6 Sant Pere Claver- Fundació Sanitària, Barcelona, Spain; 7 Centre for Biomedical Research Network on Mental Health (CIBERSAM), Instituto de Salud Carlos III, Barcelona, Spain; Harvard University, UNITED STATES

## Abstract

A common reaction experienced by family members of patients with psychosis is grief for the loss of their healthy relative. Importantly, high levels of perceived loss have been related to the manifestation of high expressed emotion (EE), which includes the negative attitudes expressed by relatives toward an ill family member. However, the mechanisms underlying the relationship between relatives’ perceived loss and EE attitudes in the early stages of psychosis are still not fully understood. In this regard, attachment theory has been suggested as a useful framework for understanding this link. The current study aimed to examine: (1) whether relatives’ perceived loss was associated with relatives’ EE dimensions (i.e., criticism and emotional over-involvement (EOI)), and (2) whether such associations were mediated by relatives’ attachment dimensions (i.e., anxiety and avoidance). Seventy-eight relatives of patients with early psychosis completed the Mental Illness Version of the Texas Inventory of Grief for the assessment of loss reactions. Attachment dimensions and EE attitudes were assessed by the Psychosis Attachment Measure and the Family Questionnaire, respectively. Findings indicated that relatives’ perceived loss was associated with EE dimensions. Relatives’ attachment anxiety, but not avoidance, mediated the relationship of perceived loss with both criticism and EOI. Findings highlight the importance of examining the role of relatives’ attachment characteristics for understanding how perceptions of loss might impact the manifestation of EE attitudes in the early stages of psychosis. Family interventions aimed at assisting relatives to improve their management of negative emotional reactions to loss are fundamental to prevent impairing loss reactions and the entrenchment of high-EE attitudes.

## Introduction

A prominent reaction described by family members of patients with psychosis is grief [1–3]. This grief results from a deep sense of loss: loss of the healthy relative, loss of specific hopes and aspirations for the relatives’ future, and loss of the pre-existing relationship between the relative and the patient [4–6]. Perceived loss has been found to have negative implications for relatives’ physical and psychological health, and also affects interactions with their ill family member [7, 8]. However, grief is not necessarily a pathological phenomenon, but rather a natural process of adapting to or accommodating a changed reality [9].

Relatives’ perceived loss has been mostly examined in schizophrenia caregivers [10–12]. However, the few studies examining relatives’ loss reactions in the early stages of psychosis indicated that high levels of perceived loss are already present in early phases of the illness [13–15]. Moreover, high levels of loss appraisals are related to the manifestation of high expressed emotion (EE) attitudes (i.e., criticism and emotional over-involvement (EOI); [13–16]). Patterson et al. [14, 15] found that high levels of perceived loss were related to high EOI attitudes, whereas low levels of perceived loss were linked to high criticism. It seems that relatives’ perception of loss could motivate high EOI attitudes with the aim of reestablishing what has been lost [14, 15]. However, coercive criticism has also been theoretically interpreted as a coping mechanism to deal with the pain of loss [17]. As argued by Patterson et al. [14, 15], this interpretation is consistent with attachment theory, which states that coercive angry or critical attitudes are a natural response to any perceived loss, with the goal to bring the person “back into line” through an activation of attachment responses [18–20]. Since the perception of loss may be a major driver of high EE attitudes [21, 22], it would be relevant to identify the mediating mechanisms by which perceived loss influences the manifestation of EE in early psychosis relatives. In this regard, attachment theory has been highlighted as a useful framework for understanding how loss appraisals may contribute to the development of EE [14, 15, 23]. Specifically, relatives’ attachment styles have received theoretical attention as potential underlying mechanisms in the association between perceived loss and EE attitudes [23].

Attachment theory has become one of the foremost paradigms for understanding the grieving process after the loss of a significant person [24–26]. Insecure attachment has been proposed as a major risk factor for complications in adaptation to loss [27–28]. Regarding the dimensions underlying attachment style, attachment anxiety and avoidance [29–30], it seems that anxious attachment is more consistently associated with elevated grief symptoms than avoidant attachment after the loss of a loved one [31–32]. Moreover, anxious/ambivalent attachment has been related to increased parental grief in response to a family member’s mental illness [33].

Attachment theory indicates that threats and stressors to an attachment bond, such as illness or loss, tend to activate the attachment system [30–34]. Indeed, persons with high levels of attachment anxiety or avoidance tend to rely on secondary attachment strategies [35–36], either hyperactivating or deactivating their attachment system for regulating threat-related distress. Hyperactivating strategies observed in anxious individuals cause them to amplify negative emotional responses to threats. In contrast, avoidant individuals attempt to inhibit emotional reactions with the goal of minimizing activation of the attachment system [30, 34, 37]. Thus, anxiously attached individuals’ tendency to intensify negative emotions would involve a heightened risk of distress after an actual or perceived loss, whereas avoidant individuals’ tendency to suppress negative emotions would promote an absence of conscious grieving [24, 37, 38].

According to Bowlby [20], parents develop their caregiving behaviors in consonance with their attachment systems and associated working models [39–42]. For instance, avoidant individuals’ lack of interpersonal engagement and negative working models of others may also translate into a lack of caregiving behaviors, probably driven by deactivating strategies across both attachment and caregiving systems [43, 44]. Conversely, anxious individuals, who adopt a hyperactivating attachment strategy [43–45]. show a pattern of hyperactivated caregiving, which is intrusive or overinvolved, poorly timed, and burdensome [30, 34]. The parallelism between these caregiving behaviors and those exhibited by high EE individuals suggests that relatives’ insecure attachment styles may influence their caregiving behaviors, contributing to high EE levels under acute stress [46]. Therefore, insecure attachment has been hypothesized to contribute to the development of critical or EOI parenting styles [47, 48]. Specifically, attachment anxiety has been related to intrusive, overinvolved, and controlling forms of caregiving [42, 44, 49, 50], as well as with the use of authoritarian and dominant behaviors in high anxiety situations [51, 52]. Altogether, this emphasizes attachment theory as a potential framework for understanding how relatives’ attachment history influences the development of EE [53].

The relationship between relatives’ attachment styles and perceived loss in the early stages of psychosis has not yet been explored. Similarly, no studies have directly considered the possible mediating role of insecure attachment styles in the association between relatives’ perceived loss and high EE attitudes. This study addressed these issues in a sample of relatives of early psychosis participants by: 1) examining whether relatives’ perceived loss is associated with EE dimensions (i.e., criticism and EOI); and 2) testing whether such associations are mediated by relatives’ dimensions of attachment (i.e., anxiety and avoidance). Despite the lack of previous studies, it was hypothesized based on the reviewed literature that relatives’ attachment anxiety would play a mediating role between perceived loss and EOI, as well as between perceived loss and criticism.

## Materials and methods

### Participants

The study included 78 relatives of early psychosis participants, 48 of At-Risk Mental State (ARMS) and 30 of First-Episode (FEP) participants. They were recruited in the Sant Pere Claver-Early Psychosis Program conducted in Barcelona, Spain [54]. Participating relatives were those who had the most regular contact and/or the most significant relationship with the patient. ARMS patients had to meet ARMS criteria as assessed by the Comprehensive Assessment of At-Risk Mental States [55] and/or the Schizophrenia Proneness Instrument Adult- Version [56]. FEP patients met DSM-IV-TR criteria [57] for any psychotic disorder or affective disorder with psychotic symptoms as established by the Structured Clinical Interview for DSM-IV [58].

All relatives provided written informed consent. The project was developed in accordance with the Code of Ethics of the World Medical Association (Declaration of Helsinki). Ethical approval was granted by the Ethics Committee of the Unió Catalana d’Hospitals (Comitè d’Ètica d’Investigació Clínica (CEIC); number 09–40) and by the Ethics Committee of the Universitat Autònoma de Barcelona (Comissió d'Ètica en l'Experimentació Animal i Humana (CEEAH); number 2679).

### Measures

Relatives completed the Mental Illness Version of the Texas Inventory of Grief (MIV-TIG) [2] for the assessment of loss reactions. The MIV-TIG is a self-reported questionnaire that contains 24 items that measure relatives’ initial response to the loss of a family member’s mental health and the current feelings about their perceived loss. Responses were rated on a five-point Likert scale from ‘completely true’ to ‘completely false’. The Cronbach’s alpha of the instrument was excellent in this sample (0.95).

Attachment was assessed with the Psychosis Attachment Measure (PAM) [59], a self-reported adult attachment questionnaire referring to self-identified thoughts, feelings and behaviors in interpersonal relationships. The PAM contains 16 items that assess the two dimensions of adult attachment, anxiety and avoidance. Responses are made on a four-point Likert scale ranging from ‘not at all’ to ‘very much’. Reliability for these subscales was acceptable in this sample (Anxiety = 0.71; Avoidance = 0.68).

Finally, relatives completed the Family Questionnaire (FQ) [60], a well-established instrument to measure EE. The FQ consists of 20 items equally distributed into two subscales (EOI and criticism) and scored on a four-point Likert scale ranging from ‘never/very rarely’ to ‘very often’. Reliability for the two subscales in this sample was 0.84 for EOI and 0.87 for criticism.

### Statistical analysis

Pearson correlations were calculated to explore the association of relatives’ perceived loss with EOI and criticism, as well as association of these variables with the two attachment dimensions. The effect size of the correlations was interpreted following Cohen’s [61] guidelines (correlations of 0.10 indicate small effect sizes, 0.30 indicate medium effect sizes, and 0.50 indicate large effect sizes). Parallel multiple mediation analyses were performed using PROCESS [62]. These analyses examined the unique mediating effect of attachment anxiety and avoidance on the significant associations found between perceived loss and the EE dimensions. For each model, attachment anxiety and avoidance were entered simultaneously as mediators. The 95% bias-corrected confidence intervals were generated using bootstrapping with 10,000 resamples. Indirect effects were considered significant when the 95% bias-corrected confidence intervals did not include zero.

### Results

Most relatives were parents who lived with the patient. Over half of them were females, specifically patients’ mothers (see Table 1 for details about relatives' socio-demographic characteristics). For the sake of completeness, descriptive data for all relatives’ measures are presented in Table 2.

**Table 1:** Descriptive data on socio-demographic characteristics of early psychosis relatives (n = 78).

	n (%)
**Age** *(mean*, *SD)*	51.05 (9.7)
**Gender**	
Males	25 (32.1)
Females	53 (67.9)
**Ethnicity**	
Caucasian-white	66 (84.6)
Other^a^	12 (15.4)^a^
**Occupation**	
Unemployed/unoccupied	34 (43.6)
Employed	44 (56.4)
**Marital Status**	
Single	4 (5.1)
Married or analogous	50 (64.1)
Separated/divorced/widowed	24 (30.8)
**Relationship to patient**	
Father	19 (24.4)
Mother	47 (60.2)
Other^b^	12 (15.4)
**Living with patient**	
Yes	65 (83.3)
No	13 (16.7)
**Frequency of contact**^c^	
Between 1 and 14h a week	28 (35.9)
Between 15 and 27h a week	14 (17.9)
≥ 28h a week	28 (35.9)

^a^ Other ethnicity was comprised by Latin Americans = 5 (6.4), Asians = 2 (2.6), Eastern Europeans = 3 (3.8) and Arabs = 2 (2.6).

^b^ Other relationship was comprised by Siblings = 10 (12.8), Grandparent = 1 (1.3) and Stepfather = 1 (1.3).

^c^ Information about frequency of contact was available only for n = 72.

**Table 2:** Descriptive data on perceived loss, attachment and expressed emotion (N = 78).

	Mean	SD	Possible score range	Observed score range
**Perceived loss (MIV-TIG)**	63.44	20.95	24–120	24–116
**Attachment (PAM)**				
Anxious Attachment	0.90	0.43	0–3	0–2.38
Avoidant Attachment	1.22	0.45	0–3	0.38–2.25
**Expressed Emotion (FQ)**				
Emotional Over-Involvement	24.33	5.85	10–40	11–37
Criticism	20.63	6.02	10–40	11–37

MIV-TIG, Mental Illness Version of the Texas Inventory of Grief; PAM, Psychosis Attachment Measure; FQ, Family Questionnaire.

Firstly, we examined whether relatives of ARMS and FEP participants differed on levels of perceived loss. No significant differences emerged between them (*t*(76) = -1.17; *p* = 0.25; *d* = 0.27). Consequently, ARMS and FEP relatives were grouped together in the remaining analyses.

Relatives’ perceived loss was significantly related to both EOI (r = 0.49, p <0.001) and criticism (r = 0.27, p<0.05). Following Cohen [61], effect sizes were of a medium and small magnitude, respectively.

As seen in Table 3, relatives’ attachment anxiety showed significant associations with relatives’ perceived loss and both EE dimensions. All effect sizes were of medium magnitude. In contrast, relatives’ attachment avoidance was significantly associated with relatives’ perceived loss but was not associated with the EE dimensions.

**Table 3:** Pearson correlations of attachment dimensions with perceived loss and expressed emotion (N = 78).

	Attachment-Anxiety	Attachment-Avoidance
**Perceived loss (MIV-TIG)**	0.38***	0.30**
**Expressed Emotion (FQ)**		
Emotional Over-Involvement	0.46***	0.22
Criticism	0.38***	0.21

**p≤0.01

***p≤ 0.001.

MIV-TIG, Mental Illness Version of the Texas Inventory of Grief; FQ: Family Questionnaire.

Note: Medium effect sizes in bold.

Table 4 displays the results of the parallel multiple mediation analyses using relatives’ perceived loss as the independent variable. Two models were tested (one for EOI and one for criticism), with the two attachment dimensions entered simultaneously as mediators. The total, direct, and indirect effects are shown in Table 4. The specific indirect effect of attachment anxiety was significant in the models for both EOI and criticism indicating that anxious attachment mediated the association of perceived loss with both EE factors. Note that the direct relationship of perceived loss and criticism no longer remained significant when the indirect pathway through anxious attachment was included. Avoidant attachment was not a significant mediator of the association between relatives’ perceived loss and EE dimensions.

**Table 4:** Mediation analyses examining the indirect effects of perceived loss on EOI and criticism via anxious and avoidant attachment.

			95% Bias-corrected Confidence Interval	
	Raw Parameter Estimate	SE	Lower	Upper
**Emotional Over-Involvement (FQ)**				
Total Effect	0.136*	0.028	0.080	0.191
Direct Effect	0.098*	0.030	0.039	0.157
Total Indirect Effect	0.038*	0.016	0.012	0.078
Indirect Effect via Anxiety	0.034*	0.014	0.013	0.071
Indirect Effect via Avoidance	0.003	0.009	-0.012	0.026
**Criticism (FQ)**				
Total Effect	0.077*	0.032	0.014	0.140
Direct Effect	0.035	0.034	-0.033	0.103
Total Indirect Effect	0.042*	0.019	0.012	0.091
Indirect Effect via Anxiety	0.033*	0.016	0.009	0.076
Indirect Effect via Avoidance	0.009	0.011	-0.007	0.037

*95% Confidence Interval does not include zero.

Note: Results are based on 10,000 bias-corrected bootstrap samples.

## Discussion

To the best of our knowledge, this is the first study examining the relationship among relatives' perceived loss, attachment styles, and EE, as well as the mediating role of relatives’ attachment dimensions in the association between perceived loss and EE, in the early stages of psychosis. Results demonstrated that relatives’ perceived loss was associated with EE attitudes as well as with relatives’ attachment anxiety and avoidance. These findings point out the importance of considering the role of relatives’ perceived loss along with other psychological mechanisms for understanding the ontogenesis of EE in the family environment. In addition, the current study indicated that relatives’ attachment anxiety, but not attachment avoidance, mediated the relationship between perceived loss and EE, both for criticism and EOI attitudes. Altogether, these results emphasize the importance of considering the role of loss in the development of EE attitudes and that relatives’ attachment style is relevant to understand how appraisals of loss might impact the formation and/or expression of EOI and criticism in the critical period of the emergence of the disorder.

Consistent with our predictions, relatives’ perceived loss was positively associated with both EOI and criticism. These results are in consonance with theoretical suggestions from the EE literature that relatives’ perception of loss may be a primary driver of high EE attitudes [21, 22]. The positive association between perceived loss and relatives’ EOI attitudes supports previous findings by Patterson et al. [14, 15] in relatives of early psychosis patients. However, in contrast to Patterson et al. [14, 15], the present study also found a positive association between perceived loss and relatives’ criticism. This finding is consistent with attachment theory that indicates that coercive criticism is not an uncommon response to perceived loss as an attempt to restore what has been “lost” through the activation of attachment responses [18, 19, 20].

**Fig 1:**
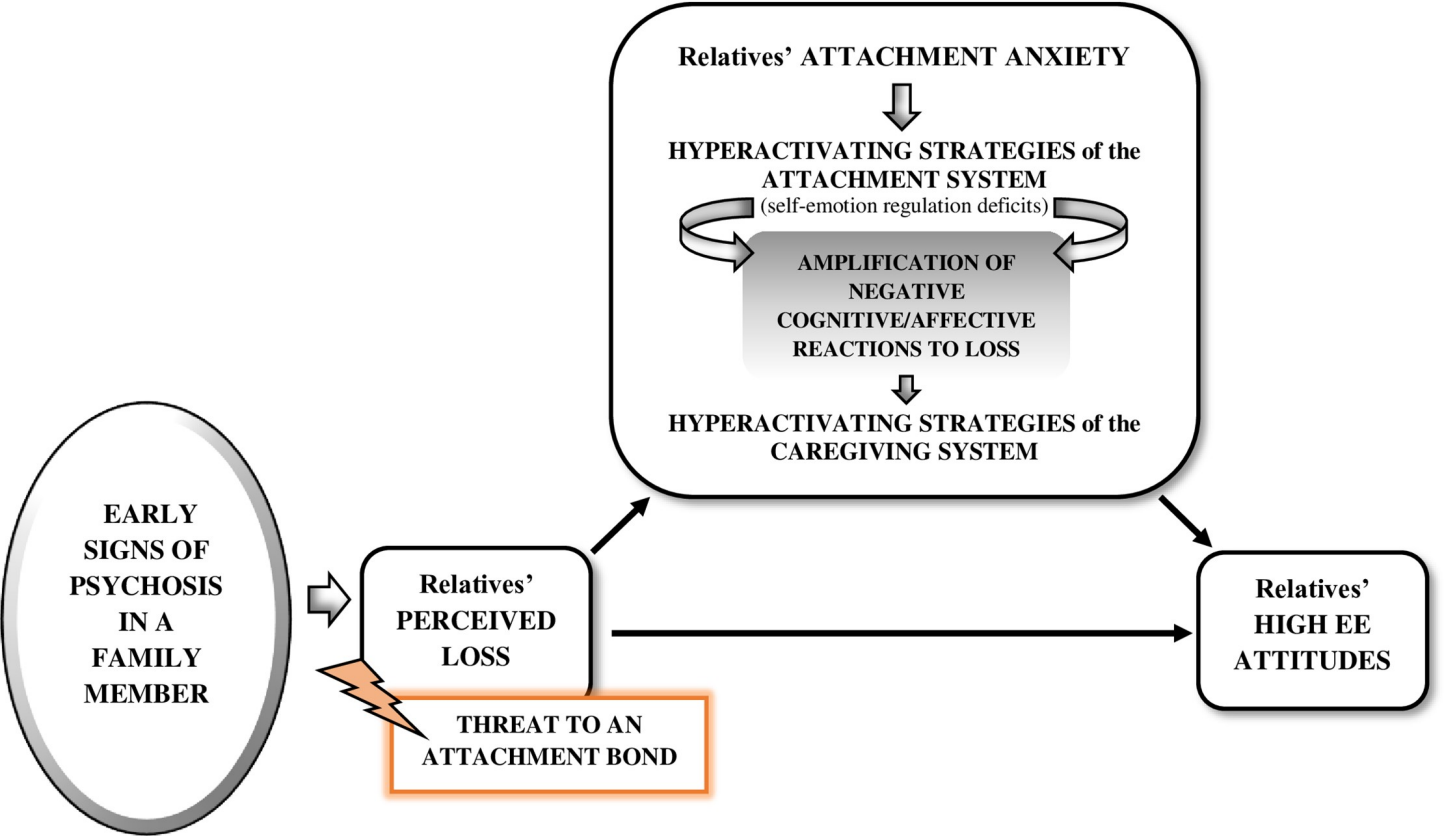
Attachment anxiety as a mechanism linking relatives’ perceived loss and EE. Proposed mediational model of the relationship between relatives’ perceived loss and high EE attitudes (i.e., criticism and EOI) in anxiously attached relatives. Adapted from Patterson (2013).

Anxious attachment in relatives, but not avoidant attachment, mediated the association of the perception of loss with both EOI and criticism. As hypothesized, this differential role of attachment styles might be explained by the specific distress regulation strategies characterizing anxious and avoidant attachment. Anxious individuals respond to appraisals of loss with hyperactivating strategies of the attachment system, which impairs their capacity to regulate negative emotions and thus contributes to an intensification of distress [30, 34, 37]. Moreover, ineffective emotional regulation strategies may make relatives feel overwhelmed by personal distress, which may in turn interfere with the normative functioning of the caregiving behavioral system [30, 34]. Theoretically, the caregiving system is activated by cues generated from care recipients in times of need [20], but it can also be activated by caregivers’ appraisals of danger or threat, which are directly influenced by caregivers’ own attachment system [40, 41]. Specifically, attachment anxiety has been associated with high levels of proximity and compulsive caregiving that are inconsistent and lack sensitivity [42, 63]. Following Canterberry and Gillath [43], this type of caregiving behaviors could reflect the hyperactivation of the caregiving system. Therefore, anxious individuals, who rely on hyperactivating strategies in their attachment system, seem to adopt this strategy with their caregiving system [43–45]. Drawing on previous theoretical suggestions based on the interplay between the attachment and caregiving systems [45, 52], our findings suggest that relatives’ perceived loss might trigger the concurrent hyperactivation of the attachment and caregiving systems in anxiously attached relatives. The inability to regulate the negative emotions provoked by perceived loss may lead to excessive ruminations about the loss, amplifying the negative affective/cognitive reactions. This would contribute to the hyperactivation of the caregiving system and lead to EE-like intrusive, overinvolved, or even critical patterns of caregiving, presumably driven by an attempt to restore the pre-illness situation and to mitigate the loss perceived (Fig 1). This explanation is partly consistent with previous suggestions (e.g., [14, 15]), even though the contribution of the caregiving behavioural system as the mechanism through which EE attitudes manifest had not been considered. Specifically, Patterson [23] proposed a theoretical model in which family illness leads to an activation of carers’ and patients’ attachment behaviors. Therefore, relatives increased high EE attitudes should be conceived as an initial normative reaction.

Previous work has linked attachment anxiety to caregiving behaviors that clearly resemble aspects of EOI behaviors (i.e., overinvolved, intrusive, controlling and/or compulsive forms of caregiving; [42, 44, 49, 50]). This has even led to the interpretation of EOI as a type of anxious attachment/caregiving behavior [64, 65]. In addition, attachment anxiety has also been related to the expression of critical attitudes through the use of authoritarian [52], aggressive [66, 67], dominant [51], and demanding behaviors in high anxiety situations [68]. Therefore, relatives’ insecure attachment could contribute to the development of either critical or EOI parenting styles [47, 48] and these two dimensions of EE could be considered as “special forms of attachment/caregiving” that are likely to lead to dysfunctional patterns of communication and problem solving [65].

Interestingly, an unexpected positive correlation between perceived loss and attachment avoidance was observed. It is well recognized that the tendency to suppress negative emotions encourages an absence of conscious grieving [24, 38, 69]. Nevertheless, some studies have found an association between attachment avoidance and severe grief symptoms after the loss of a loved one [70–72]. These findings have been interpreted as indicating that strategies of emotional deactivation might collapse under conditions of high cognitive load produced by highly stressful situations, thereby causing avoidant individuals to be less able to suppress separation thoughts. Therefore, the powerful threat of loss experienced by relatives of early psychosis individuals might weaken the suppression of the negative emotional experience of loss in relatives with avoidant attachment.

Some limitations of the present study must be considered. First, the cross-sectional design limits drawing causal conclusions, which can only be determined by further longitudinal studies. Second, it is important to note that attachment and EE-related constructs are dynamic processes influenced by bidirectional and interactional patterns within familiar relationships [73, 74]. Future studies should consider how the clinical features and attachment styles of care recipients contribute to the manifestation of high-EE attitudes within the family environment. Finally, it should be noted that although the FQ has been recognized as a reliable and valid measure to assess EE [60], it has not yet shown enough predictive validity for relapse in both chronic and early psychosis samples (e.g., [75, 76]). Therefore, further research is required to examine its predictive relevance in the course of the disorder.

In conclusion, this study shows that relatives’ perception of loss is significantly associated with EE-criticism and EE-EOI attitudes in the early stages of coping with psychosis, and that relatives’ attachment anxiety is a mediator of the association between perceived loss and the manifestation of EE attitudes in early psychosis relatives. These results have important clinical applications. Considering the negative implications of perceived loss for relatives’ psychological health and the potential etiological role of perceived loss on the development of high EE attitudes, family interventions should specifically take into consideration the psychological aspects related to loss and the grief process experienced by relatives during the at-risk and onset stages of psychosis. This emphasizes the importance to go beyond educating relatives about psychosis (e.g., [77]) by implementing treatments in which the provision of information is adapted to caregivers’ needs and allows emotional processing [78]. Consequently, this would help relatives to gain a better understanding of their own psychological situation. In addition, these findings highlight the need of tailoring interventions to relatives' attachment needs. This would include assessing relatives' attachment patterns, since the specific subtypes of insecure attachment style will provide guidance to moving forward in the therapeutic process, for example in building the working alliance or setting relevant treatment goals [79]. Early psychosis is a critical period in which relatives’ appraisals and attitudes are forming and might be more malleable; therefore, it is fundamental to support relatives in adjusting to the grief process with the aim of preventing unresolved loss reactions and the entrenchment of high-EE attitudes over time.
